# The A·T(rWC)/A·T(H)/A·T(rH) ↔ A·T*(rw_WC_)/A·T*(w_H_)/A·T*(rw_H_) mutagenic tautomerization *via* sequential proton transfer: a QM/QTAIM study

**DOI:** 10.1039/c8ra01446a

**Published:** 2018-04-10

**Authors:** Ol'ha O. Brovarets', Kostiantyn S. Tsiupa, Dmytro M. Hovorun

**Affiliations:** Department of Molecular and Quantum Biophysics, Institute of Molecular Biology and Genetics, National Academy of Sciences of Ukraine 150 Akademika Zabolotnoho Str. 03680 Kyiv Ukraine dhovorun@imbg.org.ua; Department of Molecular Biotechnology and Bioinformatics, Institute of High Technologies, Taras Shevchenko National University of Kyiv 2-h Akademika Hlushkova Ave. 03022 Kyiv Ukraine

## Abstract

In this study for the first time we have revealed by QM and QTAIM calculations at the MP2/aug-cc-pVDZ//B3LYP/6-311++G(d,p) level of QM theory the novel routes of the mutagenic tautomerization of three biologically important A·T DNA base pairs – reverse Watson–Crick A·T(rWC), Hoogsteen A·T(H) and reverse Hoogsteen A·T(rH) – followed by their rebuilding into the wobble (w) A·T*(rw_WC_), A·T*(w_H_) and A·T*(rw_H_) base mispairs by the participation of the mutagenic tautomers of the DNA bases (denoted by asterisk) and *vice versa*, thus complementing the physico-chemical property of the canonical A·T(WC) Watson–Crick DNA base pair reported earlier (Brovarets' *et al.*, *RSC Adv.*, 2015, **5**, 99594–99605). These non-dissociative tautomeric transformations in the classical A·T(rWC), A·T(H) and A·T(rH) DNA base pairs proceed similarly to the canonical A·T(WC) DNA base pair *via* the intrapair sequential proton transfer with shifting towards major or minor grooves of DNA followed by further double proton transfer along the intermolecular H-bonds and are controlled by the plane symmetric and highly stable transition states – tight ion pairs formed by the A^+^ nucleobase, protonated by the N1/N7 nitrogen atoms, and T^−^ nucleobase, deprotonated by the N3H imino group. Comparison of the estimated populations of the tautomerised states (10^−21^ to 10^−14^) with similar characteristics for the canonical A·T(WC) DNA base pair (10^−8^ to 10^−7^) leads authors to the conclusion, that only a base pair with WC architecture can be a building block of the DNA macromolecule as a genetic material, which is able for the evolutionary self-development. Among all four classical DNA base pairs, only A·T(WC) DNA base pair can ensure the proper rate of the spontaneous point errors of replication in DNA.

## Abstract 2

We discovered tautomeric wobbling of the classical A·T DNA base pairs. This data evidence, that only a base pair with Watson–Crick architecture can be a building block of the DNA macromolecule as a genetic material, which is able for the evolutionary self-development.

## Graphical abstract

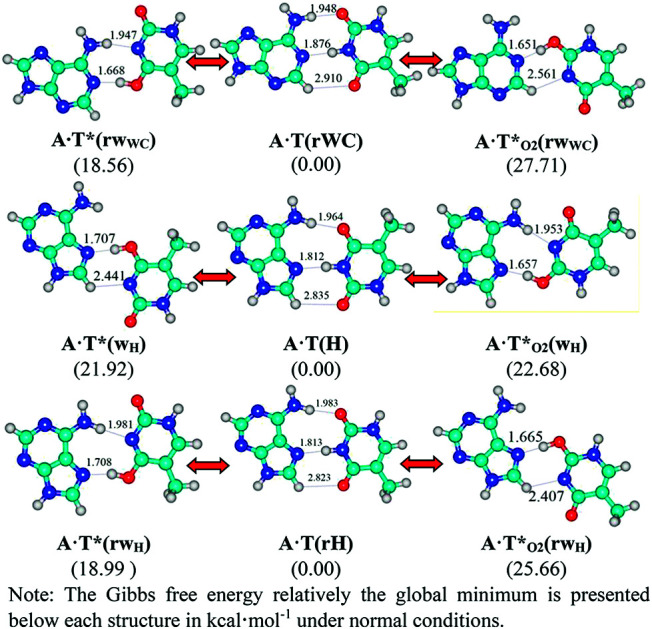

## Introduction

Clarification of the microstructural mechanisms of the mutagenic tautomerization of the DNA base pairs is a classical problem of molecular biophysics, biochemistry and structural biology, which remain topical up to now.^1–5^ Literature analysis shows that the so-called tautomeric hypothesis formulated by Watson and Crick,^1^ soon after their discovery of the spatial architecture of DNA – a macromolecule that is the carrier of the genetic information,^2^ represents itself the most vivid theoretical platform for the conduction of these studies. At that time, this hypothesis became a real breakthrough in the understanding of the nature of the origin of the spontaneous point mutations – transitions and transversions^5^ – and also involvement in this biologically important phenomenon of the prototropic tautomerism of the DNA bases.^6,7^

Advances in technology eventually led to numerous as experimental investigations,^8–13^ in particular X-ray analysis^8,9^ and NMR, in particular relaxation dispersion, measurements,^10–13^ so theoretical examinations^14–19^ of this discovery. However, these results do not clarify the physico-chemical mechanisms of the arising of the rare or mutagenic tautomeric forms of the DNA bases^20–23^ (here and below marked by an asterisk).

It was established for sure that generally accepted mechanism of the double proton transfer (DPT) along intermolecular H-bonds in the Watson–Crick (WC) (so-called Löwdin's mechanism),^24–29^ wobble (w) base pairs,^30,31^ biologically important A·G,^32^ A·C*,^33^ G*·T,^34^ C·T,^35^
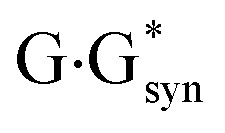
,^36^ A*·A_syn_,^37^
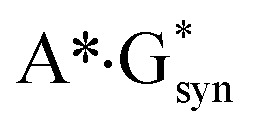
,^38^ H·C,^39,40^ H·H^39,40^ and H·A^39,42^ base mispairs and also in the protein–DNA complexes^26,43,44^ can't be considered as the source of the mutagenic tautomers formations due to the dynamical instability of the terminal complexes containing mutagenic tautomers of the DNA bases.^26–29,31–42,44^

For the first time, we have proposed a novel theoretical approach to the elucidation of the microstructural mechanisms of the incorporation and replication errors arising at the DNA replication due to the intrinsic ability of the purine·pyrimidine (A·T, G·C, G·T and A·C), purine·purine (A·A and G·G) and pyrimidine·pyrimidine (C·C and T·T) DNA base mispairs to perform WC ↔ w tautomeric transitions *via* the sequential proton transfer (PT).^45–53^ It was revealed that all these non-dissociative tautomerisations are controlled by the highly stable, highly polar and zwitterionic transition states of the type (protonated base)·(deprotonated base). These interconversions are accompanied by a significant rebuilding of the base mispairs with Watson–Crick architecture into the mismatches wobbled towards minor or major grooves of DNA. Moreover, it was established that these tautomerisation reactions occur non-dissociatively and are accompanied by the consequent replacement of the unique patterns of the intermolecular specific interactions along intrinsic reaction coordinate (IRC).

Thus, in particular, it was found out that the A·T(WC) Watson–Crick DNA base pair exists simultaneously in three other biologically important hypostasis^45^ – short-lived wobble A*·T(w) (population = 5.4 × 10^−8^), 
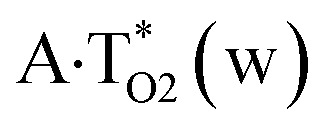
 (9.9 × 10^−9^) and A·T*(w) (2.5 × 10^−10^) H-bonded mismatches, containing mutagenic tautomers of the nucleotide bases. Their forced separation by the DNA-polymerase machinery into the monomers with necessity generates mutagenic tautomers of the DNA bases, which are long-lived structures causing spontaneous point mutations – transitions and transversions.^53–55^

Presented approach clarifies the microstructural mechanisms of the mutations induced by the classical mutagens, in particular 2-aminopurine, for which frequencies agree well with the experimental data.^56–61^

The aim of the current study is to extend the approach launched in our previous work for the canonical DNA base pairs^45^ to the other classical A·T DNA base pairs – reverse Watson–Crick A·T(rWC), Hoogsteen A·T(H) and reverse Hoogsteen A·T(rH).

At this point, the question arises according the urgency of this investigation.

First, the A·T(rWC), A·T(H) and A·T(rH) DNA base pairs have a remarkable biological meaning (see works^62–79^ and the bibliography cited therein). Second, as of today, the mutagenic tautomerization of these biologically important pairs has not even mentioned in the literature. Thirdly, we are interested in the investigation of the evolutionary aspect of the problem, in particular, why *Nature* chose precisely Watson–Crick DNA base pairs for the construction of the genetic material, among which the A·T(WC) DNA base pair is the most evolutionarily distant, since it was the first to appear evolutionary.^6,80,81^

So, in this regard, we can make an assumption that exactly the A·T(WC) base pair provides necessary frequency of the spontaneous point replication errors in DNA, which lies in the range of 10^−9^ to 10^−11^ per nucleotide, incorporated during one replication cycle.^82,83^

Such statement of the problem except merely academic value has also practical assignment, *e.g.* for the biomolecular electronics, which are used for the DNA-based carriers of the digital information,^84,85^ since it allows, in principle, to understand how the complementary bases should be modified in order to suppress the tautomeric instability of their pair. This is extremely important for increasing of the accuracy of such molecular devices.^86^

As a result of the systematic quantum-mechanical calculations, we managed to establish the microstructural mechanisms of the mutagenic tautomerisation of the studied A·T DNA base pairs and to reach the conclusion about a unique place of the canonical Watson–Crick A·T(WC) DNA base pair among them. Only this base pair able to provide the necessary rate of the spontaneous point mutations, which, as it is well known, are the source of the genome self-development.^6,80–83^

## Computational methods

Geometries of the investigated DNA base pairs and transition states (TSs) of their mutual tautomeric transformations, as well as their harmonic vibrational frequencies have been calculated at the B3LYP/6-311++G(d,p) level of theory,^87–91^ using Gaussian'09 package^92^ followed by the IRC calculations in the forward and reverse directions from each TS using Hessian-based predictor-corrector integration algorithm.^93^ Applied level of theory has proved itself successful for the calculations of the similar systems.^94–96^ A scaling factor that is equal to 0.9668 (ref. 97–100) has been applied in the present work for the correction of the harmonic frequencies for all DNA base pairs and TSs of their tautomeric transitions. We have confirmed the local minima and TSs, localized by Synchronous Transit-guided Quasi-Newton method,^101^ on the potential energy landscape by the absence or presence, respectively, of the imaginary frequency in the vibrational spectra of the complexes. We applied standard TS theory for the estimation of the activation barriers of the tautomeric transformations.^102^ Electronic energy calculations have been performed at the MP2/aug-cc-pVDZ level of theory.^103,104^

The Gibbs free energy *G* for all structures was obtained in the following way:

1 *G* = *E*_el_ + *E*_corr_,

where *E*_el_ – electronic energy, while *E*_corr_ – thermal correction.

The time *τ*_99.9%_ necessary to reach 99.9% of the equilibrium concentration of the reactant and product in the system of reversible first-order forward (*k*_f_) and reverse (*k*_r_) reactions can be estimated by the formula:^102^2
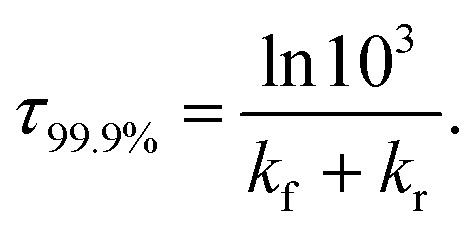


The lifetime *τ* of the formed mismatches has been calculated using the formula (1)/*k*_r_, where the values of the reverse *k*_r_ and forward *k*_f_ rate constants for the tautomerisation reactions were obtained as:^102^3
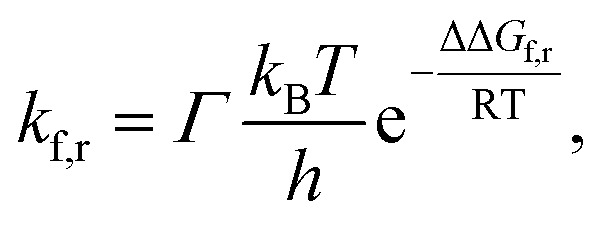
where quantum tunneling effect has been accounted by Wigner's tunneling correction,^105^ which has been successfully used for the DPT reactions:^33–42^4
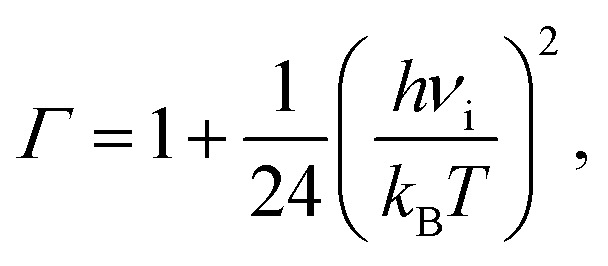
where *k*_B_ – Boltzmann's constant, *h* – Planck's constant, ΔΔ*G*_f,r_ – Gibbs free energy of activation for the tautomerisation reaction in the forward (f) and reverse (r) directions, *ν*_i_ – magnitude of the imaginary frequency associated with the vibrational mode at the TSs.

Electronic interaction energies Δ*E*_int_ have been calculated at the MP2/6-311++G(2df,pd) level of theory as the difference between the total energy of the base pair and energies of the monomers and corrected for the basis set superposition error (BSSE)^106,107^ through the counterpoise procedure.^108,109^

Bader's quantum theory of atoms in molecules (QTAIM),^110–115^ using program package AIMAll,^116^ was applied to analyse the electron density distribution. The presence of the bond critical point (BCP), namely the so-called (3,−1) BCP, and a bond path between hydrogen donor and acceptor, as well as the positive value of the Laplacian at this BCP (Δ*ρ* > 0), were considered as criteria for the H-bond formation.^117,118^ Wave functions were obtained at the level of theory used for geometry optimisation.

The energies of the AH⋯B conventional H-bonds were evaluated by the empirical Iogansen's formula:^119^5
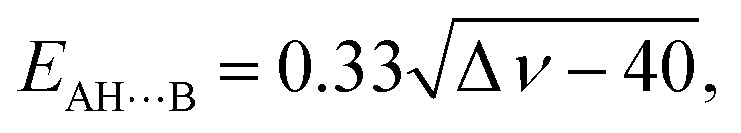
where Δ*ν* – magnitude of the frequency shift of the stretching mode of the H-bonded AH group involved into the AH⋯B H-bond relatively the unbound group. The partial deuteration was applied to minimize the effect of vibrational resonances.^120–122^

The energies of the weak CH⋯O/N H–bonds^123,124^ were calculated by the empirical Espinosa–Molins–Lecomte formula^125,126^ based on the electron density distribution at the (3,−1) BCPs of the H-bonds:

6 *E*_HB_ = 0.5 *V*(*r*),

where *V*(*r*) – value of a local potential energy at the (3,−1) BCP.

The energies of the NH⋯O H-bonds in the TSs of the DPT tautomerisations containing loosened covalent bridge have been estimated by the Nikolaienko–Bulavin–Hovorun formula:^127^

7 *E*_NH⋯O_ = −2.03 + 225*ρ*,

where *ρ* – the electron density at the (3,−1) BCP of the H-bond.

The atomic numbering scheme for the DNA bases is conventional.^128^

## Results and their discussion

**Fig. 1:**
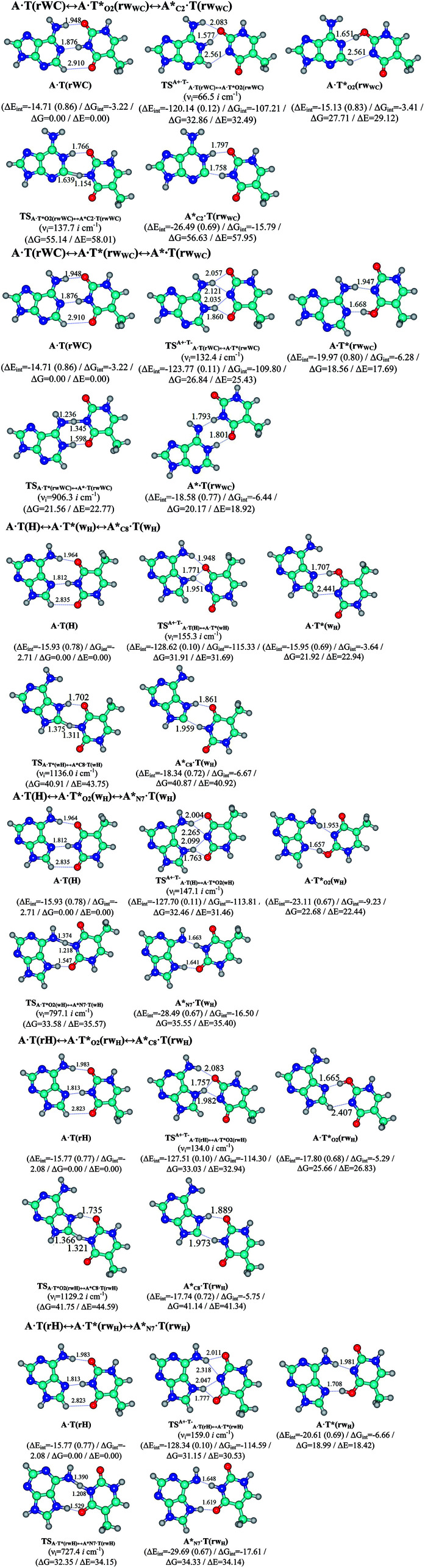
Geometrical structures of the stationary points on the pathways of the tautomerization of the classical A·T DNA base pairs into the wobble base mispairs *via* the sequential PT followed by DPT. Electronic Δ*E*_int_ (contribution of the total energy of the H-bonds) and Gibbs free Δ*G*_int_ energies of the interaction (MP2/6-311++G(2df,pd)//B3LYP/6-311++G(d,p) level of theory, in kcal mol^−1^), relative Gibbs free energies Δ*G* and electronic energies Δ*E* (in kcal mol^−1^), imaginary frequencies *ν*_i_ at the TSs of the tautomeric transitions (MP2/aug-cc-pVDZ//B3LYP/6-311++G(d,p) level of theory in the continuum with *ε* = 1 at *T* = 298.15 K) are presented below complexes in brackets. Dotted lines indicate AH⋯B H-bonds – their lengths H⋯B are presented in angstroms (for their more detailed physico-chemical characteristics see Table 2); carbon atoms are in light-blue, nitrogen – in dark-blue, hydrogen – in grey and oxygen – in red.

In this work based on the results obtained in the pioneering publication,^45^ devoted to the novel WC ↔ w mutagenic tautomerization of the canonical A·T(WC) and G·C(WC) DNA base pairs, we have investigated for the first time the microstructural mechanisms of the mutagenic tautomerisation of the three other biologically important A·T DNA base pairs^62–79^ – A·T(rWC)/A·T(H)/A·T(rH) ↔ A·T*(rw_WC_)/A·T*(w_H_)/A·T*(rw_H_) – as their intrinsically inherent property (Fig. 1, Tables 1 and 2).

**Table 1:** Energetic (in kcal mol^−1^) and kinetic (in s) characteristics of the tautomerization of the classical A·T DNA base pairs into the wobble base mispairs *via* the sequential PT followed by DPT obtained at the MP2/aug-cc-pVDZ//B3LYP/6-311++G(d,p) level of QM theory in the continuum with *ε* = 1 under normal conditions (see Fig. 1)

Tautomeric transition	*ν*_i_ a	Δ*G*^b^	Δ*E*^c^	ΔΔ*G*_TS_^d^	ΔΔ*E*_TS_^e^	ΔΔ*G*^f^	ΔΔ*E*^g^	*τ*_99.9%_ h	*τ* i	*P* j
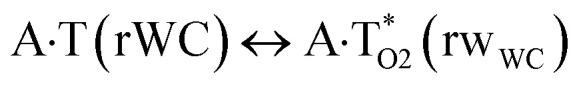	66.5	27.71	29.12	32.86	32.49	5.15	3.37	6.62 × 10^−9^	9.58 × 10^−10^	4.72 × 10^−21^
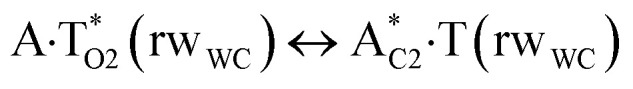	137.7	28.92	29.23	27.43	29.29	−1.49	0.06	8.83 × 10^−14^	1.28 × 10^−14^	2.91 × 10^−42^
A·T(rWC) ↔ A·T*(rw_WC_)	132.4	18.56	17.69	26.84	25.43	8.29	7.74	1.31 × 10^−6^	1.90 × 10^−7^	2.45 × 10^−14^
A·T*(rw_WC_) ↔ A*·T(rw_WC_)	906.3	1.61	1.24	3.00	5.08	1.39	3.85	1.08 × 10^−11^	1.67 × 10^−12^	1.60 × 10^−15^
A·T(H) ↔ A·T*(w_H_)	155.3	21.92	22.94	31.91	31.69	9.99	8.75	2.31 × 10^−5^	3.35 × 10^−6^	8.39 × 10^−17^
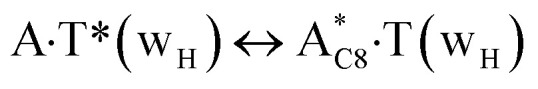	1136.0	18.95	17.97	18.99	20.81	0.04	2.84	5.56 × 10^−13^	8.04 × 10^−14^	1.06 × 10^−30^
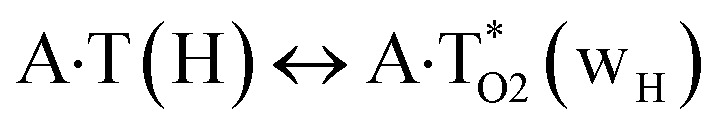	147.1	22.68	22.44	32.46	31.63	9.78	9.19	1.62 × 10^−5^	2.34 × 10^−6^	2.32 × 10^−17^
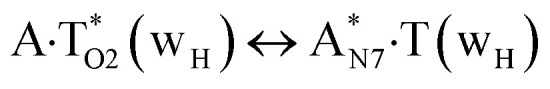	797.1	12.87	12.96	10.90	13.14	−1.97	0.18	2.54 × 10^−14^	3.68 × 10^−15^	8.45 × 10^−27^
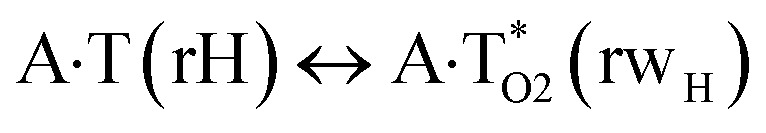	134.0	25.66	26.83	33.03	32.94	7.38	6.11	2.81 × 10^−7^	4.07 × 10^−8^	1.52 × 10^−19^
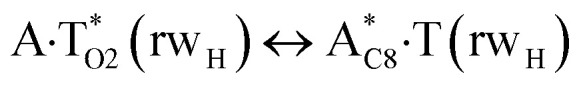	1129.2	15.49	14.51	16.09	17.76	0.60	3.25	1.43 × 10^−12^	2.08 × 10^−13^	6.61 × 10^−31^
A·T(rH) ↔ A·T*(rw_H_)	159.0	18.99	18.42	31.15	30.53	12.17	12.11	9.13 × 10^−4^	1.32 × 10^−4^	1.18 × 10^−14^
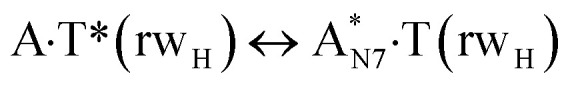	727.4	15.34	15.73	13.36	15.73	−1.98	0.01	3.86 × 10^−14^	5.58 × 10^−15^	6.65 × 10^−26^

a The imaginary frequency at the TS of the tautomeric transition, cm^−1^.

b The Gibbs free energy of the product relatively the reactant of the tautomeric transition (*T* = 298.15 K).

c The electronic energy of the product relatively the reactant of the tautomeric transition.

d The Gibbs free energy barrier for the forward tautomeric transition.

e The electronic energy barrier for the forward tautomeric transition.

f The Gibbs free energy barrier for the reverse tautomeric transition.

g The electronic energy barrier for the reverse tautomeric transition.

h The time necessary to reach 99.9% of the equilibrium concentration between the reactant and the product of the tautomerisation reaction, s.

i The lifetime of the product of the tautomerisation reaction, s.

j The thermal population of the tautomerised structures, which is situated on the right in the first row of the table.

**Table 2:** Electron-topological, geometrical and energetic characteristics of the intermolecular H-bonds in the investigated DNA base pairs and TSs of their tautomerization into the wobble base mispairs *via* the sequential PT followed by DPT obtained at the B3LYP/6-311++G(d,p) level of QM theory (*ε* = 1) (see Fig. 1)

Complex	AH⋯B H-bond	*ρ* a	Δ*ρ*^b^	100*ε*^c^	*d*_A⋯B_ d	*d*_H⋯B_ e	∠AH⋯B^f^	*E*_AH⋯B_ g	*μ* h
A·T(rWC)	N6H⋯O2	0.024	0.088	5.26	2.962	1.949	172.9	4.38	2.40
	N3H⋯N1	0.039	0.093	6.51	2.887	1.843	177.7	7.55	
	C2H⋯O4	0.004	0.014	3.32	3.696	2.872	132.8	0.77*	
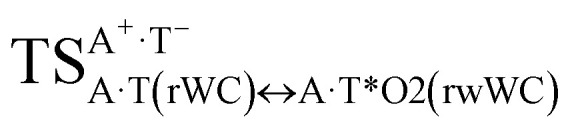	N6^+^H⋯O2^−^	0.017	0.073	8.71	2.910	2.083	136.9	2.99	9.34
	N1^+^H⋯O2^−^	0.067	0.133	1.76	2.614	1.577	159.6	10.16	
	C2^+^H⋯N3^−^	0.011	0.034	18.55	3.207	2.561	117.3	1.81*	
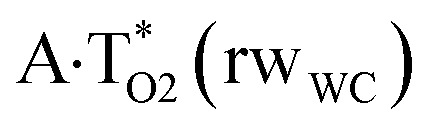	O2H⋯N7	0.058	0.100	4.73	2.682	1.665	179.7	10.35	5.10
	C8H⋯N3	0.013	0.043	2.29	3.131	2.407	123.1	2.21*	
TS_A·T*O2(rwWC)↔A*C2·T(rwWC)_	N1H⋯O2	0.040	0.125	4.34	2.761	1.766	159.5	6.91**	5.36
A*_C2_·T(rw_WC_)	N1H⋯O2	0.037	0.120	4.38	2.787	1.797	159.5	5.77	5.21
	N3H⋯C2^−^	0.061	0.033	103.30	2.840	1.758	164.3	12.53	
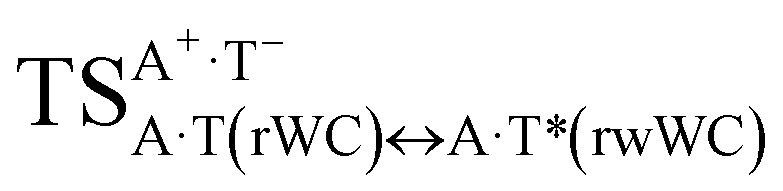	N6^+^H⋯O2^−^	0.020	0.067	12.45	3.026	2.057	155.7	2.86	6.11
	N6^+^H⋯N3^−^	0.020	0.069	13.99	2.971	2.121	138.4	2.82	
	N1^+^H⋯N3^−^	0.024	0.081	32.39	2.932	2.035	141.8	2.92	
	N1^+^H⋯O4^−^	0.034	0.098	5.50	2.805	1.860	148.0	4.45	
A·T*(rw_WC_)	N6H⋯N3	0.030	0.087	7.07	2.682	1.668	170.4	5.76	2.52
	O4H⋯N1	0.059	0.096	5.10	2.955	1.947	167.0	10.21	
TS_A·T*(rwWC)↔A*·T(rwWC)_	N1H⋯O4	0.061	0.142	3.32	2.663	1.598	179.3	11.61**	3.78
A*·T(rw_WC_)	N3H⋯N6	0.044	0.095	6.22	2.844	1.793	174.7	8.53	3.23
	N1H⋯O4	0.035	0.117	3.55	2.832	1.801	177.3	5.82	
A·T(H)	N6H′⋯O4	0.023	0.086	3.93	2.972	1.963	170.6	4.18	6.16
	N3H⋯N7	0.041	0.099	5.75	2.853	1.811	175.9	7.39	
	C8H⋯O2	0.005	0.016	7.71	3.524	2.835	121.7	0.83*	
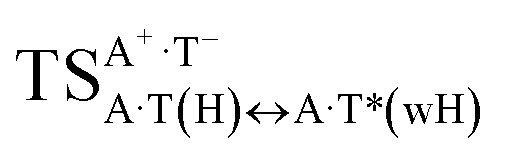	N6^+^H′⋯O4^−^	0.022	0.091	1.81	2.936	1.948	161.7	4.76	2.09
	N7^+^H⋯O4^−^	0.041	0.112	6.15	2.749	1.771	152.5	5.03	
	N7^+^H⋯N3^−^	0.029	0.097	35.39	2.784	1.951	133.5	3.27	
A·T*(w_H_)	O4H⋯N7	0.052	0.102	4.74	2.717	1.707	178.5	8.99	4.74
	C8H⋯N3	0.012	0.040	2.99	3.149	2.441	121.9	2.08*	
TS_A·T*(wH)↔A*C8·T(wH)_	N7H⋯O4	0.047	0.133	2.94	2.678	1.702	153.3	8.55**	3.56
A*_C8_·T(w_H_)	N7H⋯O4	0.031	0.109	3.24	2.810	1.861	152.7	5.00	6.08
	N3H⋯C8^−^	0.035	0.061	4.38	2.975	1.959	161.6	8.30	
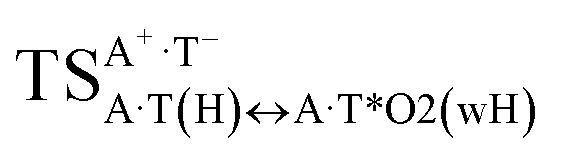	N6^+^H′⋯O4^−^	0.024	0.079	2.31	2.887	2.004	142.4	3.64	6.54
	N6^+^H′⋯N3^−^	0.014	0.049	67.29	3.218	2.265	153.9	1.84	
	N7^+^H⋯N3^−^	0.021	0.076	382.35	3.022	2.099	144.8	2.36	
	N7^+^H⋯O2^−^	0.042	0.115	3.62	2.688	1.763	143.9	5.72	
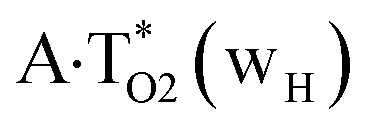	N6H′⋯N3	0.029	0.086	7.38	2.974	1.953	176.4	5.38	8.23
	O2H⋯N7	0.059	0.100	4.48	2.664	1.657	168.0	10.16	
TS_A·T*O2(wH)↔A*N7·T(wH)_	N7H⋯O2	0.067	0.152	3.15	2.615	1.547	176.3	12.95**	9.46
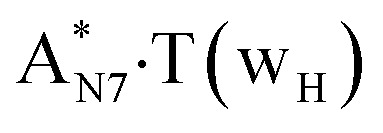	N3H⋯N6	0.060	0.092	5.58	2.743	1.663	175.7	10.97	10.35
	N7H⋯O2	0.051	0.145	3.17	2.689	1.641	176.3	8.09	
A·T(rH)	N6H′⋯O2	0.022	0.082	4.95	2.994	1.986	170.9	3.90	5.67
	N3H⋯N7	0.041	0.099	5.80	2.856	1.815	176.9	7.34	
	C8H⋯O4	0.005	0.017	7.97	3.517	2.825	121.9	0.86*	
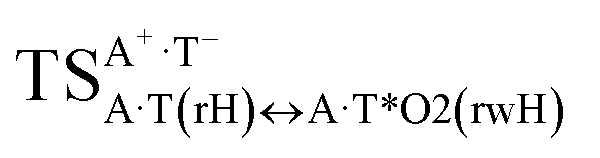	N6^+^H′⋯O2^−^	0.016	0.066	0.24	3.064	2.083	161.1	4.06	3.41
	N7^+^H⋯O2^−^	0.042	0.114	5.64	2.757	1.757	157.6	5.05	
	N7^+^H⋯N3^−^	0.028	0.098	49.28	2.758	1.982	128.1	3.02	
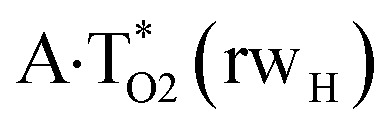	O2H⋯N7	0.058	0.100	4.73	2.682	1.665	179.7	9.20	5.10
	C8H⋯N3	0.013	0.043	2.29	3.131	2.407	123.1	2.21*	
TS_A·T*O2(rwH)↔A*C8·T(rwH)_	N7H⋯O2	0.043	0.129	3.56	2.698	1.735	151.8	7.70**	4.79
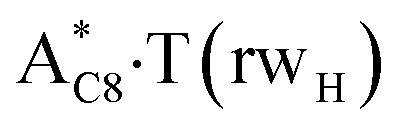	N7H⋯O2	0.029	0.104	3.92	2.829	1.889	151.4	4.61	6.47
	N3H⋯C8^−^	0.034	0.061	4.31	2.984	1.973	160.5	8.12	
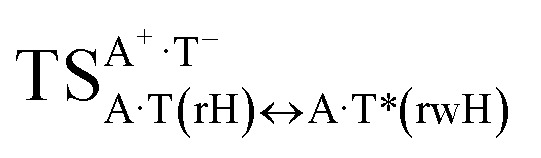	N6^+^H′⋯O2^−^	0.023	0.078	1.54	2.902	2.011	143.9	3.51	4.80
	N6^+^H′⋯N3^−^	0.013	0.045	127.55	3.263	2.318	152.9	1.65	
	N7^+^H⋯N3^−^	0.023	0.078	82.90	2.987	2.047	146.7	2.64	
	N7^+^H⋯O4^−^	0.041	0.113	4.62	2.686	1.776	141.6	5.65	
A·T*(rw_H_)	N6H′⋯N3	0.027	0.082	7.62	3.000	1.981	175.7	5.09	7.36
	O4H⋯N7	0.052	0.102	4.48	2.702	1.708	166.4	9.18	
TS_A·T*(rwH)↔A*N7·T(rwH)_	N7H⋯O4	0.070	0.151	2.34	2.603	1.529	175.7	13.76**	8.37
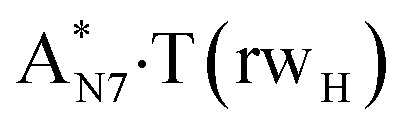	N3H⋯N6	0.062	0.090	5.55	2.731	1.648	174.5	11.26	9.42
	N7H⋯O4	0.055	0.147	2.33	2.671	1.619	175.8	8.61	

a The electron density at the (3,−1) BCP of the H-bond, a.u.

b The Laplacian of the electron density at the (3,−1) BCP of the H-bond, a.u.

c The ellipticity at the (3,−1) BCP of the H-bond.

d The distance between the A and B atoms of the of the AH⋯B H-bond, Å.

e The distance between the H and B atoms of the AH⋯B H-bond, Å.

f The H-bond angle, degree.

g Energy of the H-bond, calculated by Iogansen's,^119^ Espinose–Molins–Lecomte^125,126^ (marked with an asterisk) or Nikolaienko–Bulavin–Hovorun^127^ (marked with double asterisk) formulas, kcal mol^−1^.

h The dipole moment of the complex, *D*.

It was found that the mutagenic tautomerization of each of these classical base pairs is controlled by the two TSs, representing itself tight (electronic energy of the bases interaction ∼120–129 kcal mol^−1^) ion pairs (A^+^ nucleobase, protonated by the N1/N7 nitrogen atoms)·(T^−^ nucleobase, deprotonated by the N3H imino group) with plane symmetric (*C*_s_ symmetry) quasi-wobble structure. The term “quasi-wobble” means that these structures are no longer rWC/H/rH, but are not yet wobble. Notably, they differ from each other by the shifting direction of the T^−^ respectively A^+^ (towards major or minor groove of DNA) and also by the number of the H-bonds, which participate in their stabilization, – three or four, – one or two of them are characterized by the increased ellipticity (Fig. 1, Table 2). The latter points to the dynamic instability of these H-bonds.^31,97^ Thus, the 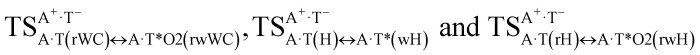 transition states, in which the T^−^ deprotonated by the N3H imino group, is shifted towards the minor groove of DNA relatively A^+^, are stabilized by the participation of three H-bonds: (A)N6^+^H⋯O2^−^(T) (2.99), (A)N1^+^H⋯O2^−^(T) (10.16) and (A)C2^+^H⋯N3^−^(T) (1.81 kcal mol^−1^); (A)N6^+^H′···O4^−^(T) (4.76), (A)N7^+^H⋯O4^−^(T) (5.03) and (A)N7^+^H⋯N3^−^(T) (3.27 kcal mol^−1^); (A)N6^+^H′···O2^−^(T) (4.06), (A)N7^+^H⋯O2^−^(T) (5.05) and (A)N7^+^H⋯N3^−^(T) (3.02 kcal mol^−1^) (their energies are presented in brackets), accordingly (Table 2). At this, in each TS only one H-bond has increased ellipticity (its value is presented in brackets) – (A)C2^+^H⋯N3^−^(T) (18.55); (A)N7^+^H⋯N3^−^(T) (35.39); (A)N7^+^H⋯N3^−^(T) (49.28), respectively (Fig. 1, Table 2).

Three other 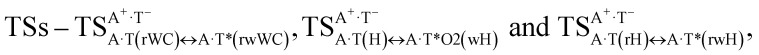 in which the T^−^ deprotonated by the N3H imino group, is shifted towards major groove of DNA relatively A^+^, are joined by the participation of the four H-bonds: (A)N6^+^H⋯O2^−^(T) (2.86), (A)N6^+^H⋯N3^−^(T) (2.82), (A)N1^+^H⋯N3^−^(T) (2.92) and (A)N1^+^H⋯O4^−^(T) (4.45 kcal mol^−1^); N6^+^H′⋯O4^−^ (3.64), N6^+^H′⋯N3^−^ (1.84), N7^+^H⋯N3^−^ (2.36) and N7^+^H⋯O2^−^ (5.72 kcal mol^−1^); N6^+^H′⋯O2^−^ (3.51), N6^+^H′⋯N3^−^ (1.65), N7^+^H⋯N3^−^ (2.64) and N7^+^H⋯O4^−^ (5.65 kcal mol^−1^). Two H-bonds have increased ellipticity for each of these TSs – (A)N6^+^H⋯N3^−^(T) (13.99) and (A)N1^+^H⋯N3^−^(T) (32.39); N6^+^H′⋯N3^−^ (67.29) and N7^+^H⋯N3^−^ (382.35); N6^+^H′⋯N3^−^ (127.55) and N7^+^H⋯N3^−^ (82.90), accordingly (Fig. 1, Table 2).

Values of the Gibbs free energies of activation of the processes of the dipole-active tautomerization of the investigated A·T DNA base pairs are quite high and lie within the range 27–33 kcal mol^−1^ under normal conditions (Fig. 1, Table 1).

The 
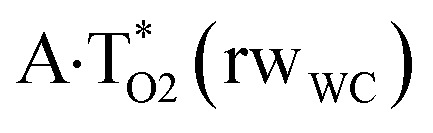
, A·T*(rw_WC_), A·T*(w_H_), 
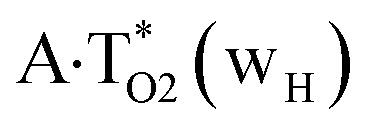
, 
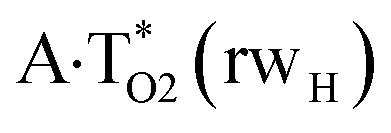
 and A·T*(rw_H_) base mispairs, which are the products of the mutagenic tautomerization of classical A·T DNA base pairs, represent themselves wobble structures with plane symmetric architecture (*C*_s_ symmetry), stabilized by two anti-parallel intermolecular H-bonds. They are noticeably more stable than the starting A·T(rWC), A·T(H) and A·T(rH) DNA base pairs and have quite high relative energies, lying in the range 19–28 kcal mol^−1^, and hence – insignificant population (≤1.2 × 10^−14^ under normal conditions). It is interesting to note, that these wobble base mispairs are figuratively speaking “terminal stations” on the way of the mutagenic tautomerization of the investigated DNA base pairs, since they do not tautomerise further (Fig. 1, Tables 1 and 2).

Really, the 
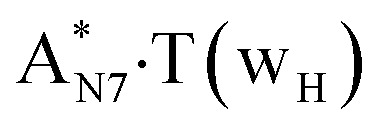
 and 
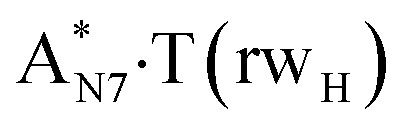
 complexes, which are formed from the 
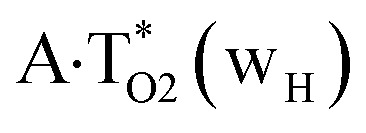
 and A·T*(rw_H_) base pairs *via* the DPT, respectively, return without any barrier into the initial pairs due to the asynchronous DPT along the intermolecular H-bonds *via* the TS_A·T*O2(wH)↔A*N7·T(wH)_ and TS_A·T*(rwH)↔A*N7·T(rwH)_, accordingly. The same situation also takes place for the complex by the participation of the yilidic form^20,39,129^ of A – 
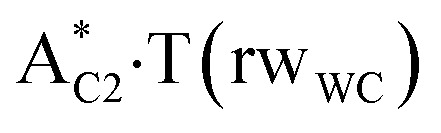
. Two other 
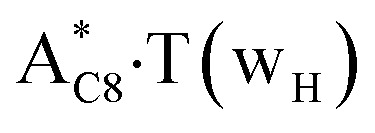
 and 
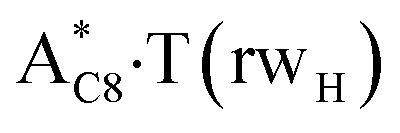
 complexes involving yilidic forms of the A DNA base, which are formed from the A·T*(w_H_) and 
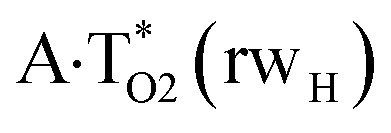
 by the asynchronous DPT along the intermolecular H-bonds, one of which (A)C8H⋯N3(T) H-bond is non-canonical,^123,124^ are short-lived (∼0.1 ps), dynamically-unstable systems. Low-frequency intermolecular normal vibrations, lying in the range 20–83 cm^−1^, could not develop during their lifetimes. From the other side, the lifetime of the A*·T(rw_WC_) complex (1.7 ps), which is formed from the A·T*(rw_WC_) pair by the asynchronous DPT along the intermolecular H-bonds, is significantly less than the time (10^−9^ s)^27,28^, spent by the DNA-polymerase for the forced dissociation of the complementary pairs of the DNA bases into the monomers. As a result, this complex “slips out of its hands” and canonical tautomeric status of the A DNA base does not change (Table 1).

Base pairs remain plane symmetric structures during the entire PT and DPT tautomerization processes along the IRC. The methyl group of the T DNA base does not change its orientation during these tautomerization processes *via* the PT and DPT. Moreover, the heterocycles of the A and T DNA bases remain planar, despite their ability for the out-of-plane bending^130–133^ (Fig. 1).

Interestingly, that the total energy of the intermolecular H-bonds only partially contributes to the electron energy of the monomers interactions among all without any exceptions H-bonded structures investigated in this work (see Fig. 1). In particular, in the TSs of mutagenic tautomerization, which are ion pairs, contribution of the H-bonds into the energy of their stabilization consist only 10–12% in comparison with the background of strong electrostatic (Coulomb) interactions. In other complexes it is much higher – from 67 to 86% (Fig. 1). These regularities agree well with the previously reported data for the other H-bonded pairs of nucleotide bases.^31–42^

## Conclusions

So, revealed microstructural mechanisms of the mutagenic tautomerization of the A·T DNA base pairs provide the generation of the mutagenic tautomers of only one among two DNA bases, in particular T DNA base, within the pair of bases. However, this generation is much more slower in comparison with the classical A·T(WC) DNA base pair and does not provide adequate population of the mutagenic tautomers (10^−9^ to 10^−11^).

Finally, these results lead us to a conclusion, which is very interesting from an evolutionary point of view:^6,80,81^ among all classical pairs of the DNA bases only the Watson–Crick A·T(WC) DNA base pair can pretend on the role of the building block of the genetic material – DNA macromolecule with antiparallel strands, able for the self-development during large time intervals.

## Conflicts of interest

There are no conflicts to declare.

## Supplementary Material
